# Pathophysiological Mechanisms of UVB Irradiation in the Skin: Inflammation, Oxidative Stress and Neuroimmune Interactions

**DOI:** 10.3390/pathophysiology33040076

**Published:** 2026-10-08

**Authors:** Lucas Sobral de Rossi, Renata M. Martinez, Geovana Martelossi-Cebinelli, Soraia Mendes-Pierotti, Willian H. Chinen, Jonathan Ratko, Bianca Mattioli Trambaioli, Josiane A. Vignoli, Sandra R. Georgetti, Marcela M. Baracat, Fabiana T. M. C. Vicentini, Waldiceu A. Verri, Rubia Casagrande

**Affiliations:** 1 Department of Pharmaceutical Sciences, State University of Londrina, Londrina 3371-2475, PR, Brazilrenatamimartinez@uel.br (R.M.M.);; 2 Department of Immunology, Parasitology and General Pathology, State University of Londrina, Londrina 86.057-970, PR, Brazil; 3 Department of Biochemistry and Biotechnology, State University of Londrina, Londrina 86.057-970, PR, Brazil; 4 School of Pharmaceutical Sciences of Ribeirao Preto, University of Sao Paulo, Ribeirao Preto 14040-903, SP, Brazil

**Keywords:** UVB radiation, oxidative stress, neuroimmune interactions

## Abstract

Ultraviolet B (UVB) irradiation, with wavelengths ranging from 290 to 320 nm, represents a biologically active fraction of solar irradiation that reaches the Earth’s surface and exerts significant effects on human skin. Due to its high energy, UVB penetrates the epidermis and directly interacts with cellular components, particularly DNA, leading to the formation of photoproducts such as cyclobutane pyrimidine dimers. In parallel, UVB exposure induces the generation of reactive oxygen species, promoting oxidative stress and damage to lipids, proteins, and genetic material. These events activate key intracellular signaling pathways, including NF-κB and MAPKs, resulting in the production of pro-inflammatory mediators that amplify cutaneous inflammation. In addition to classical inflammatory and oxidative mechanisms, emerging evidence highlights the role of neuroimmune interactions in the skin, involving crosstalk between nerve endings, neuropeptides, and immune cells, which contributes to the regulation of local immune responses following UVB exposure. The integration of these processes plays a central role in the pathophysiology of UVB-induced skin damage and in the development of skin diseases. This review will discuss the molecular and cellular mechanisms underlying the cutaneous response triggered by UVB irradiation, with emphasis on inflammation, oxidative stress, and neuroimmune communication. Additionally, current and emerging therapeutic strategies based on pathophysiology mechanisms, including antioxidant agents, anti-inflammatory compounds, and photoprotective approaches, will be discussed to highlight potential avenues for improved prevention and treatment of UVB-induced skin damage.

## 1. UVB Radiation

**Figure 1:**
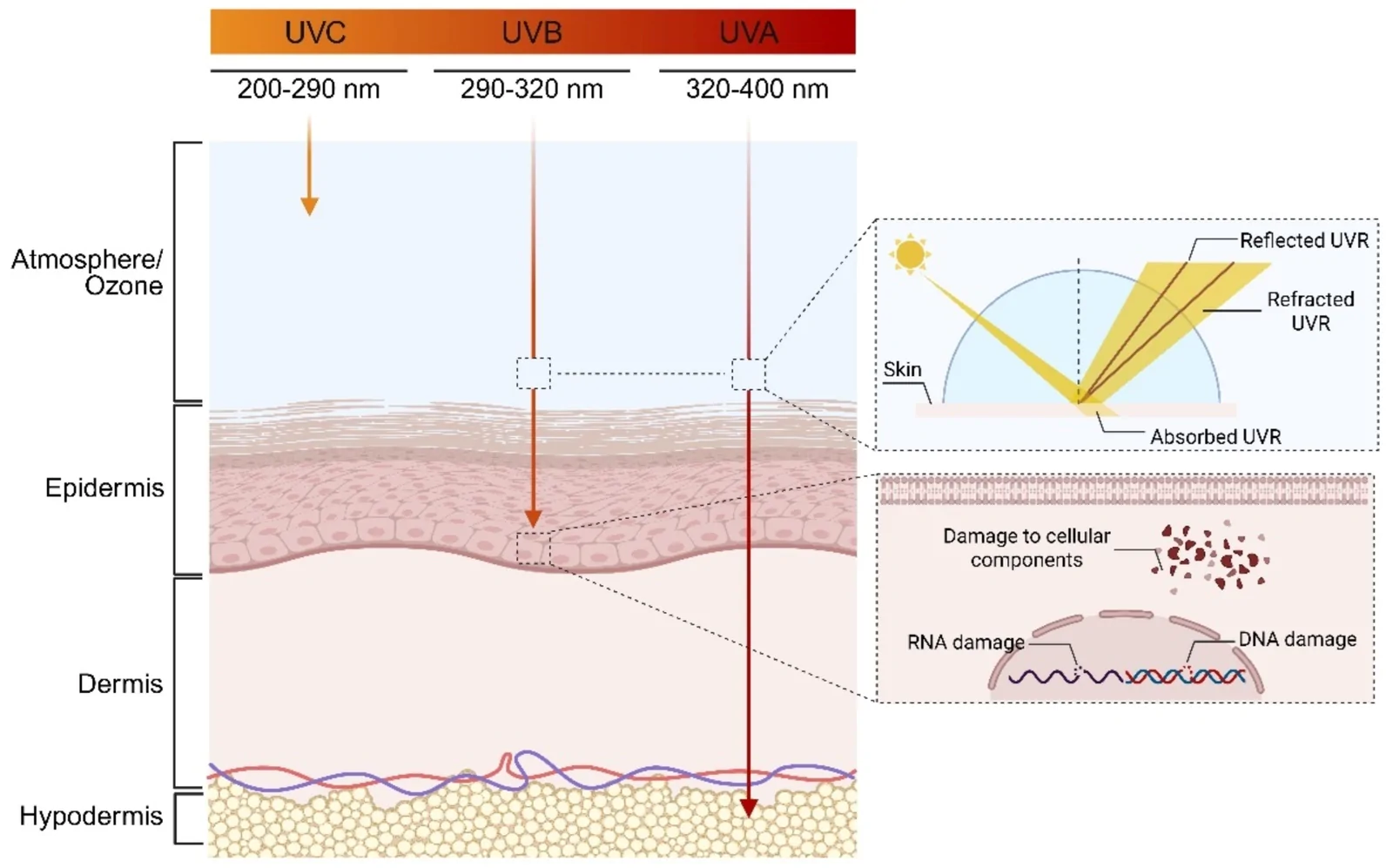
Characteristics and interaction of ultraviolet radiation with the atmosphere and human skin. Ultraviolet radiation (UVR) is divided into UVC (200–290 nm), UVB (290–320 nm), and UVA (320–400 nm) according to wavelength. UVC is effectively absorbed by the atmosphere and ozone layer, whereas part of UVB and most UVA reach the Earth’s surface. After reaching the skin, UVR can be reflected, refracted, or absorbed by tissue chromophores. UVB exhibits relatively low penetration and acts predominantly within the epidermis and superficial dermis, whereas UVA penetrates more deeply through the dermis and may reach the subcutaneous tissue. Absorption of UVR by cellular chromophores can trigger photochemical reactions that result in damage to DNA, RNA, proteins, and other cellular components, contributing to UVR-induced cutaneous injury. Abbreviations: UVR, ultraviolet radiation. Created in BioRender. Verri, W.A. (2026) https://BioRender.com/v4q6fyt.

Ultraviolet radiation (UVR) is the major exogenous environmental factor responsible for skin damage. UVR is divided into three wavelength ranges: UVA (320–400 nm), UVB (290–320 nm), and UVC (200–290 nm) (Figure 1). Each wavelength range contributes to cutaneous damage through distinct mechanisms. UVA has a high penetration capacity, reaching the epidermis, dermis, and even the subcutaneous tissue. In contrast, UVB has a lower penetration capacity, concentrating its harmful effects mainly within the epidermis and upper dermis [1].

UVR reaching the Earth’s surface consists predominantly of UVA, accounting for approximately 95%, whereas only about 5% is composed of UVB. Upon reaching the skin, part of this radiation is reflected, refracted, or absorbed. At the molecular level, chromophores are responsible for photon absorption [2]. Following absorption, these molecules enter electronically excited states and may interact with components of the biological environment, leading to the formation of products capable of inducing tissue damage. Both UVA and UVB radiation promote the formation of these products and contribute to cutaneous injury. However, despite representing a smaller fraction of solar UVR, UVB plays a more significant role in photocarcinogenesis because it directly damages DNA, RNA, proteins, and other cellular components [3].

The differences among the effects produced by the various UVR wavelength ranges are directly related to their physical properties. Wavelength is one of the main parameters determining the interaction of electromagnetic radiation with matter, and even within the ultraviolet spectrum, biological activity varies considerably according to this parameter. This characteristic, together with the absorption of UVB by several molecules present in the skin, accounts for its lower penetration depth compared with UVA [4]. UVB is estimated to penetrate approximately 0.1 mm into the skin, whereas UVA can reach depths of approximately 0.8 mm [4].

The amount of UVB available for biological exposure is also strongly determined by its interaction with the Earth’s atmosphere. As solar radiation passes through the atmosphere, all UVC and approximately 90% of UVB radiation are absorbed by components such as ozone, water vapor, oxygen, and carbon dioxide, whereas UVA is less attenuated [5]. Therefore, although UVB represents only a small fraction of the UVR that reaches the Earth’s surface, its intensity varies considerably according to environmental and geographical conditions.

The main factors determining UVR levels at the Earth’s surface include solar elevation, time of day, season, latitude, altitude, ozone concentration, cloud cover, and reflection from terrestrial surfaces [5]. Solar elevation is particularly important because, when the Sun is lower on the horizon, radiation travels a greater distance through the atmosphere, thereby increasing attenuation. This effect is especially pronounced at wavelengths below 320 nm, making UVB more sensitive than UVA to variations in solar position [6]. Consequently, the highest levels of UVR exposure generally occur during the hours around solar noon. Altitude also influences exposure, as the reduced atmospheric thickness at higher elevations decreases radiation attenuation [5].

In addition to natural sources, UVB radiation can be artificially generated for experimental and therapeutic applications. Fluorescent lamps, xenon arc lamps, and LED-based technologies can generate radiation within specific regions of the ultraviolet spectrum [4,6]. However, simply classifying a source as “UVB” does not fully describe its properties, since different devices may exhibit distinct spectral distributions within the same wavelength range. Therefore, characterization of the spectral distribution is essential for adequately defining the nature of the radiation used in photobiological studies [6].

From a radiometric perspective, another fundamental aspect is the amount of radiation incident on the tissue. Irradiance corresponds to the radiant power incident on a given area and is generally expressed in W/m^2^ or W/cm^2^, whereas its integration over time corresponds to radiant exposure, frequently referred to as dose in photobiological studies and expressed in J/m^2^ or J/cm^2^ [6]. Accordingly, radiant exposure is determined by the irradiance of the source and the irradiation time [4].

Thus, the effects of UVB radiation on the skin result not only from the total amount of incident energy but also from the interaction among its spectral characteristics, intensity, exposure duration, environmental conditions, and the optical and molecular properties of the irradiated tissue. These physical characteristics provide the basis for understanding the cellular and molecular mechanisms triggered following cutaneous exposure to UVB.

## 2. Methods

This review was written based on the author’s experience and a critical interpretation of the literature on the pathophysiological mechanisms associated with cutaneous UVB exposure. A literature search was conducted in PubMed between March and August 2026. Search terms were combined according to the main topics covered in this review, which were terms related to UVB radiation and skin damage (“ultraviolet B”, “UVB”, “ultraviolet radiation”, “skin photodamage”, “photoaging”), inflammation and oxidative stress (“inflammation”, “oxidative stress”, “reactive oxygen species”, “NF-κB”, “MAPK”, “NLRP3”), skin immune cells (“keratinocytes”, “fibroblasts”, “Langerhans cells”, “mast cells”, “neutrophils”), neuroimmune interactions (“neuroimmune”, “neuropeptides”, “CGRP”, “substance P”, “nociceptors”, “TRP channels”), and photoprotective and therapeutic strategies (“photoprotection”, “antioxidants”, “anti-inflammatory”, “therapeutic strategies”, “nanocarriers”, “resolvins”).

No lower publication-year limit or language restriction was applied, allowing the inclusion of relevant classical studies when appropriate. Experimental in vitro and in vivo studies, human studies, relevant reviews, and representative studies addressing the main topics of the manuscript were considered for inclusion. Studies outside the scope of the review, as well as duplicate or redundant publications, were excluded. Institutional sources and reference databases were used when necessary to complement specific conceptual or technical information. Articles were selected according to their relevance and representativeness to provide an integrated and critical overview of the mechanisms involved in the cutaneous response to UVB radiation.

## 3. Structure and Immune System of the Skin

The skin constitutes the primary interface between the organism and the external environment and performs essential functions in the maintenance of homeostasis. In addition to limiting excessive water and electrolyte loss, it contributes to thermoregulation, sensory perception, and protection against physical, chemical, and biological agents. This protective function does not rely exclusively on a mechanical barrier but rather results from the integration of structural, chemical, microbiological, immunological, and neuronal components. Therefore, the skin should be regarded as a structurally complex and immunologically active organ capable of detecting environmental changes and coordinating responses aimed at preserving tissue integrity [7,8].

**Figure 2:**
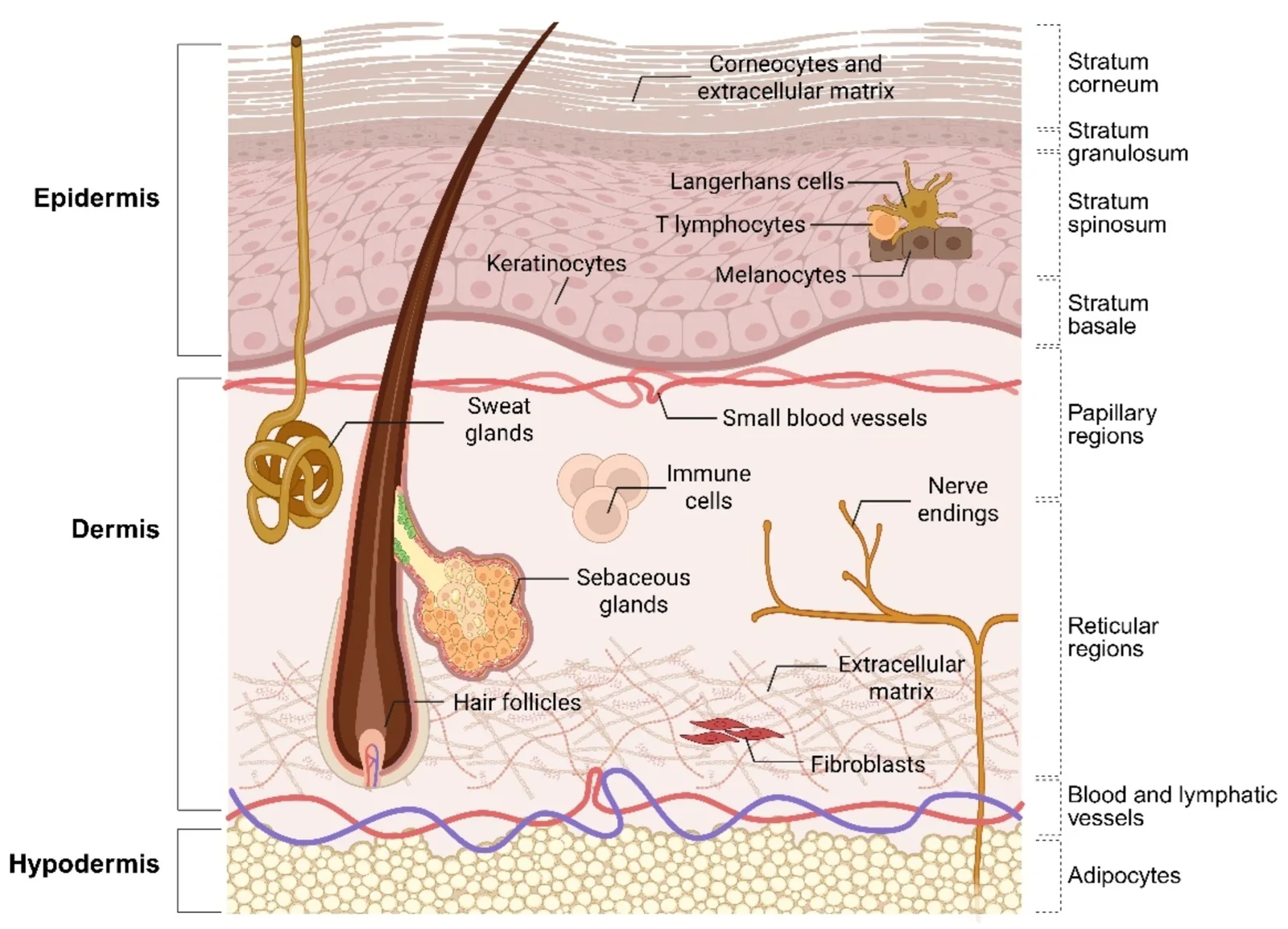
Structural, cellular, and immunological organization of human skin. The skin is organized into three major compartments: the epidermis, dermis, and hypodermis. The epidermis is a stratified epithelium composed predominantly of keratinocytes distributed across the stratum basale, stratum spinosum, stratum granulosum, and stratum corneum, and also contains melanocytes, Langerhans cells, and T lymphocytes. The dermis is subdivided into papillary and reticular regions and contains fibroblasts, extracellular matrix, resident and recruited immune cells, blood and lymphatic vessels, nerve endings, hair follicles, sebaceous glands, and sweat glands. The hypodermis is composed predominantly of adipocytes and is associated with larger blood and lymphatic vessels. Together, these structural, vascular, immune, and neuronal components form an integrated tissue network that supports barrier function, immune surveillance, sensory perception, and coordinated responses to environmental stimuli. Created in BioRender. Verri, W.A. (2026) https://BioRender.com/v4q6fyt.

Structurally, the skin can be divided into three main compartments: the epidermis, dermis, and subcutaneous tissue (Figure 2). The epidermis is the outermost layer and constitutes the primary interface with the external environment. Beneath the epidermis lies the dermis, which is composed of connective tissue and characterized by the presence of extracellular matrix, blood and lymphatic vessels, nerve endings, skin appendages, and diverse cellular populations. Finally, the subcutaneous tissue, located beneath the dermis, contains a high proportion of adipocytes and performs functions related to energy storage, thermal insulation, and the production of various bioactive mediators [7]. The organization of these compartments also determines the distribution of immune cells and directly influences the nature of the responses elicited by environmental stimuli.

The epidermis is a stratified epithelium composed predominantly of keratinocytes at different stages of differentiation, organized into the stratum basale, stratum spinosum, stratum granulosum, and stratum corneum. The stratum basale represents the deepest region of the epidermis and contains highly proliferative keratinocytes responsible for the continuous renewal of the epithelium. During differentiation, these cells progressively migrate toward the skin surface, passing through the stratum spinosum and stratum granulosum until they reach the stratum corneum, where they become terminally differentiated corneocytes [8,9]. This continuous differentiation process is essential for preserving the integrity of the skin surface and replacing cells that are continuously lost through cell death or mechanical friction.

The stratum corneum plays a central role in skin barrier function. It is composed of corneocytes enriched in structural proteins and surrounded by a predominantly lipid-based extracellular matrix. Its major lipid components include ceramides, cholesterol, and free fatty acids, which form organized structures between corneocytes and reduce the permeability of the skin surface. In addition, proteins of the cornified envelope, keratin filaments, and intercellular adhesion structures contribute to its mechanical resistance. Thus, the stratum corneum simultaneously acts as an “outside–in” barrier, limiting the entry of microorganisms, chemical substances, and other environmental agents, and as an “inside–out” barrier, restricting water loss [7,10].

However, barrier function is not restricted to the stratum corneum. The nucleated layers of the epidermis also contribute to permeability control through intercellular adhesion structures, including tight junctions, desmosomes, and adherents’ junctions. The integration of these structures with cytoskeletal proteins maintains keratinocyte cohesion and contributes to the mechanical strength of the tissue. Alterations in these components can impair epidermal barrier function, facilitating the entry of external agents and the development of inflammatory responses [10]. Therefore, the physical integrity of the epidermis is closely associated with the maintenance of cutaneous immune homeostasis.

Although keratinocytes perform a fundamental structural function, they are not passive cells. In addition to constituting the predominant cell population of the epidermis, they act as important immune sentinels capable of detecting alterations in the local microenvironment and initiating defensive responses. These cells express pattern-recognition receptors, including several Toll-like receptors (TLRs), and can detect both microorganism-derived molecules and signals associated with cellular damage. Following activation, keratinocytes can produce cytokines, chemokines, and antimicrobial peptides, thereby establishing communication with both resident and recruited immune cells [8,9]. Thus, keratinocytes establish an important functional link between the physical barrier and the immune response.

In addition to keratinocytes, the epidermis contains melanocytes, Langerhans cells, and populations of T lymphocytes. Melanocytes are located predominantly within the basal layer and are responsible for melanin synthesis. Melanin is transferred to neighboring keratinocytes and exerts an important protective effect against UVR. Beyond their pigmentary function, melanocytes are also capable of producing cytokines and chemokines and may therefore contribute to the regulation of the cutaneous immune microenvironment [9]. This reinforces the concept that cells traditionally regarded as structural or specialized for other functions can also contribute to cutaneous immunity.

Langerhans cells represent an important population of antigen-presenting cells resident in the epidermis. Distributed among keratinocytes, these cells continuously survey the epidermal microenvironment and are capable of capturing antigens. Upon activation, they may undergo functional changes and migrate toward lymphatic vessels and draining lymph nodes, where they participate in the activation or modulation of T-cell responses. Under homeostatic conditions, they also contribute to the maintenance of immune tolerance to self and environmental antigens [7,8]. The distribution of Langerhans cells is not random; experimental evidence indicates that these cells maintain a relatively stable spatial organization within the epidermis and adjust their distribution according to epithelial cell density, thereby promoting broad immune surveillance of the cutaneous surface [11].

Beneath the epidermis lies the dermis, a structurally more complex compartment. It can be subdivided into papillary and reticular regions and contains an extracellular matrix composed mainly of collagen and elastic fibers, together with proteoglycans and other components synthesized predominantly by fibroblasts. The papillary dermis is located immediately beneath the dermo-epidermal junction and contains a network of small blood vessels, whereas the reticular dermis is thicker, contains a greater concentration of collagen fibers, and houses structures such as hair follicles, sebaceous glands, and sweat glands [7]. The presence of blood and lymphatic vessels within this compartment also enables nutrient transport and communication among the skin, systemic circulation, and lymphoid organs.

Fibroblasts represent one of the major structural cell populations of the dermis and are responsible for the synthesis and maintenance of extracellular matrix components, including collagen, proteoglycans, and elastic fibers. However, similar to keratinocytes, fibroblasts can also exert immunological functions. In response to adequate stimuli, these cells produce cytokines, chemokines, growth factors, and other mediators capable of regulating the recruitment and activity of immune cells [9]. Therefore, bidirectional communication between epidermal keratinocytes and dermal fibroblasts contributes to the integration of responses occurring within different skin compartments.

The dermis harbors a particularly diverse immune cell population composed of dermal dendritic cells, macrophages, mast cells, T lymphocytes, and other resident populations, in addition to cells that may be recruited from the circulation during inflammatory processes. Dermal dendritic cells function as antigen-presenting cells and constitute an important link between innate and adaptive immunity. Resident macrophages, in turn, participate in the clearance of apoptotic cells and cellular debris, maintenance of tissue homeostasis, and coordination of inflammatory and reparative responses [7]. Resident lymphocytes also contribute to immune surveillance and enable rapid responses to local alterations [8].

Mast cells constitute another particularly relevant resident population within the dermis. These cells are distributed near blood vessels, skin appendages, and nerve endings, a strategic localization that enables them to rapidly respond to changes in the cutaneous microenvironment. Their granules contain several biologically active mediators, including histamine, proteases, and other components capable of modifying vascular permeability and the activity of neighboring cells. In addition, mast cells can produce cytokines, growth factors, prostaglandins, and leukotrienes, thereby contributing to both the amplification and regulation of inflammatory and immune responses [7,12]. Their anatomical proximity to blood vessels and nerve fibers also positions them as important components of cutaneous neuroimmune communication.

The integration of the different resident cell populations of the skin led to the concept of skin-associated lymphoid tissue (SALT), which defines the skin as an organized immune system composed of both professional immune cells and structural cells capable of participating in immune responses. Within this system, keratinocytes, melanocytes, fibroblasts, and endothelial cells act in concert with Langerhans cells, dermal dendritic cells, macrophages, mast cells, and lymphocytes to detect environmental alterations and coordinate mechanisms of defense, tolerance, and tissue repair [9]. This organization allows the skin to rapidly respond to potentially harmful stimuli without necessarily resulting in excessive inflammation, with the balance between defense and tolerance being essential for the preservation of tissue integrity [8].

In addition to its epithelial and immunological components, the skin is extensively innervated by sensory nerve fibers. Peripheral nerve endings are distributed predominantly throughout the dermis and extend toward more superficial layers, allowing the detection of mechanical, thermal, and chemical stimuli. However, these nerve fibers are not exclusively involved in sensory transmission. Peripheral neurons are capable of detecting mediators produced by resident and immune cells and reciprocally releasing neurotransmitters and neuropeptides capable of modulating vascular, epithelial, and immune functions [13,14].

Among the mediators involved in this communication are neuropeptides such as substance P (SP) and calcitonin gene-related peptide (CGRP), which can act on several cell types within the skin. The close anatomical association of sensory fibers with mast cells, antigen-presenting cells, blood vessels, and structural cells provides the basis for bidirectional communication between the nervous and immune systems. Thus, signals originating from tissue injury or alterations in the local microenvironment can be detected by nerve endings and converted into local responses, whereas inflammatory mediators produced by cutaneous cells can, in turn, modify neuronal activity [13,14]. Accordingly, the skin is currently recognized not only as a physical and immunological barrier but also as an important neuroimmune interface.

This structural organization is particularly relevant to the cutaneous response to UVR. Because UVB exhibits limited penetration and interacts predominantly with the epidermis, keratinocytes and other cells located within this compartment are among the first cellular components exposed to radiation [4]. Nevertheless, the resulting response is not restricted to the site of radiation absorption. Communication among keratinocytes, melanocytes, Langerhans cells, fibroblasts, resident immune cells, blood vessels, and nerve endings enables a stimulus initially confined to the epidermis to be converted into an integrated tissue response. Therefore, understanding the structural, immunological, and neuroimmune organization of the skin is essential for interpreting the inflammatory, oxidative, and cellular mechanisms subsequently triggered by UVB exposure.

## 4. Non-Neuronal Cellular and Molecular Mechanisms

### 4.1. Cellular Targets of UVB Radiation

**Figure 3:**
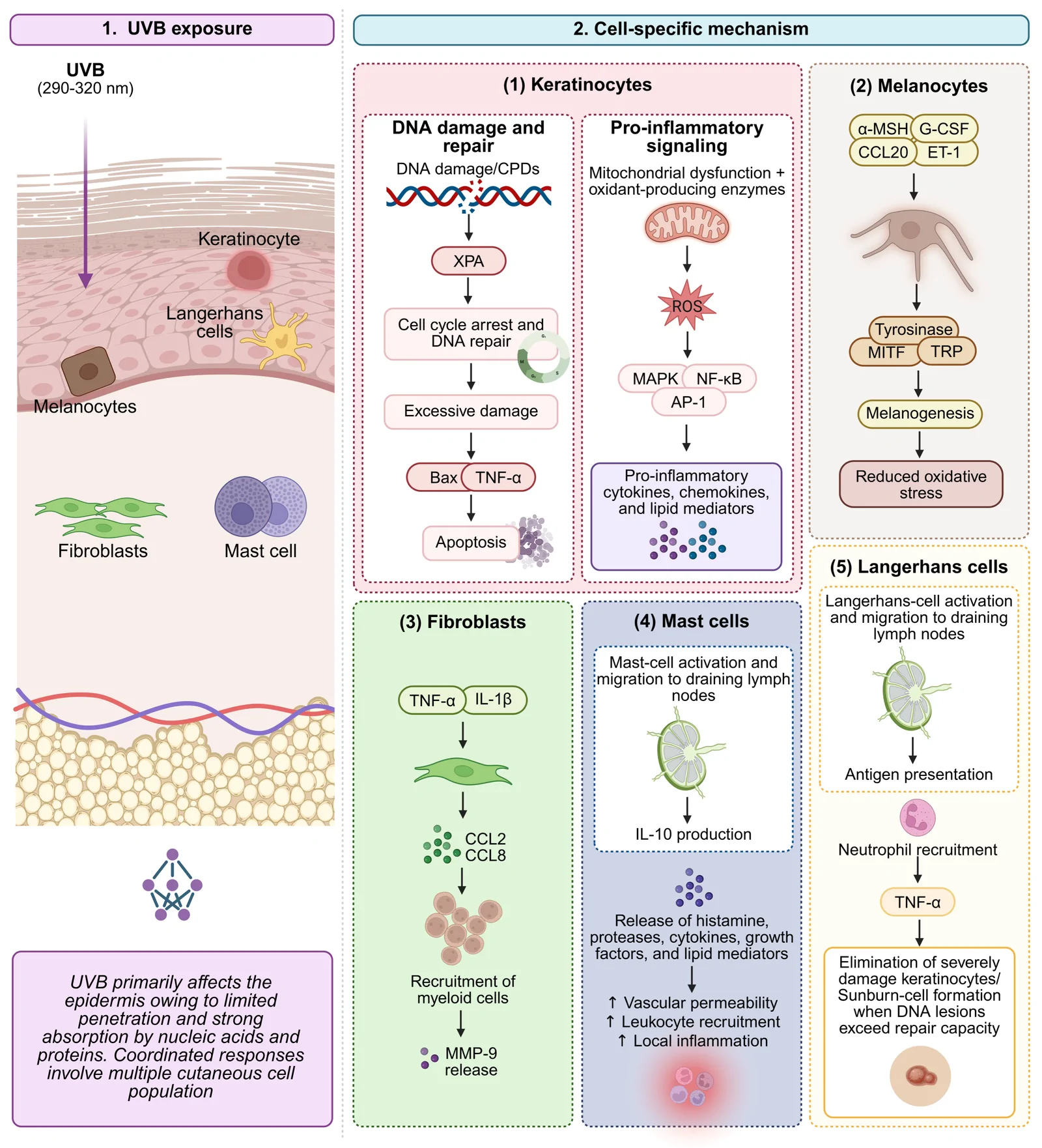
Non-neuronal mechanisms specific to each cell type involved in the cutaneous response to UVB radiation. UVB radiation (290–320 nm) acts predominantly on the epidermis, where keratinocytes represent the main cellular target, while coordinated responses involving melanocytes, fibroblasts, Langerhans cells, and mast cells propagate the effects of irradiation through different skin compartments. In keratinocytes, UVB induces direct DNA damage and the formation of CPDs, activating XPA-dependent DNA repair and cell cycle arrest. When DNA damage exceeds repair capacity, mechanisms associated with Bax and TNF-α promote apoptosis and the elimination of severely damaged cells. UVB also induces mitochondrial dysfunction and ROS generation, activating MAPK, NF-κB, and AP-1 signaling and stimulating the production of pro-inflammatory cytokines, chemokines, and lipid mediators. α-MSH, G-CSF, CCL20, and ET-1 derived from keratinocytes regulate melanocyte responses, promoting MITF-, tyrosinase-, and TRP-dependent melanogenesis and contributing to the reduction of oxidative stress. In the dermis, TNF-α and IL-1β stimulate fibroblasts to produce CCL2 and CCL8, promoting myeloid cell recruitment and MMP-9 release. Mast cell activation promotes the release of histamine, proteases, cytokines, growth factors, and lipid mediators, increasing vascular permeability, leukocyte recruitment, and local inflammation; mast cell migration to draining lymph nodes and IL-10 production further contribute to UVB-induced immunomodulation. Finally, Langerhans cells are activated and migrate to the draining lymph nodes, participating in antigen presentation and promoting neutrophil recruitment. Recruited neutrophils produce TNF-α and contribute to the elimination of severely damaged keratinocytes and the formation of sunburn-like cells when DNA damage exceeds cellular repair capacity. Together, these interconnected cellular responses contribute to the inflammatory, immunological, oxidative, pigmentary, and tissue remodeling effects of UVB radiation exposure. Abbreviations: α-MSH, alpha-melanocyte-stimulating hormone; AP-1, activator protein 1; Bax, Bcl-2-associated protein X; CCL2, C-C motif chemokine ligand 2; CCL8, C-C motif chemokine ligand 8; CCL20, C-C motif chemokine ligand 20; CPDs, cyclobutane pyrimidine dimers; ET-1, endothelin-1; G-CSF, granulocyte colony-stimulating factor; IL, interleukin; MAPK, mitogen-activated protein kinase; MITF, microphthalmia-associated transcription factor; MMP-9, matrix metalloproteinase 9; NF-κB, nuclear factor kappa B; ROS, reactive oxygen species; TNF-α, Tumor necrosis factor-alpha; TRP, tyrosinase-related protein; UVB, ultraviolet B radiation; XPA, xeroderma pigmentosum group A protein. Created in BioRender. Verri, W.A. (2026) https://BioRender.com/v4q6fyt.

The UVB radiation primarily affects the epidermis owing to its limited penetration depth and high absorption by biological chromophores, particularly nucleic acids and proteins. Although keratinocytes represent the predominant cellular target upon UVB exposure, the biological consequences of photodamage arise from coordinated responses involving multiple cutaneous cell populations, including melanocytes, fibroblasts, Langerhans cells, mast cells, and endothelial cells. Together, these cells form an integrated network that regulates inflammation, oxidative stress, immune responses, vascular changes, and tissue remodeling following UVB irradiation (Figure 3) [15].

### 4.2. Keratinocytes

Keratinocytes serve as the primary cellular targets of UVB radiation, which induces direct DNA damage through the formation of mutagenic photolesions known as cyclobutane pyrimidine dimers (CPDs). This damage alters cellular physiology, triggering responses that range from cell cycle arrest and DNA repair—governed by the Xeroderma Pigmentosum group A (XPA) protein—to apoptosis in cases of excessive injury. Upon the initiation of programmed cell death, damaged keratinocytes transform into SBC, a vital protective mechanism mediated by the activation of the pro-apoptotic gene Bax and the presence of Tumor necrosis factor-alpha (TNF-α). The efficient clearance of these cells is orchestrated by an immunological network wherein Langerhans cells regulate chemokine responses to recruit TNF-α-producing neutrophils, thereby ensuring the removal of compromised keratinocytes and preventing the accumulation of mutations that increase the risk of skin cancer [15].

In addition to direct genotoxicity, UVB exposure promotes the generation of reactive oxygen species (ROS) through mitochondrial dysfunction and activation of oxidant-producing enzymes. Elevated ROS levels activate multiple signaling pathways, including MAPKs, NF-κB, and AP-1, resulting in the production of pro-inflammatory cytokines, chemokines, and lipid mediators. Consequently, keratinocytes act not only as primary targets of UVB-induced damage but also as central regulators of the subsequent inflammatory and immunological responses in the skin [16].

### 4.3. Melanocytes

Melanocytes are specialized pigment-producing cells located within the basal layer of the epidermis. Their primary function is to synthesize melanin and transfer melanosomes to neighboring keratinocytes, thereby reducing UV-induced DNA damage. Following UVB exposure, melanocytes increase melanogenesis through activation of microphthalmia-associated transcription factor (MITF), tyrosinase, and tyrosinase-related proteins [16].

A recent study has demonstrated that melanocyte responses are strongly influenced by keratinocyte-derived mediators. UVB-damaged keratinocytes release factors such as α-melanocyte-stimulating hormone (α-MSH), ET-1, granulocyte colony-stimulating factor (G-CSF), and CCL20, which enhance melanocyte survival, stimulate melanogenesis, and reduce oxidative stress. Therefore, melanocytes contribute not only to pigmentation but also to adaptive protective responses against UVB-induced injury [16].

### 4.4. Fibroblasts

Although fibroblasts are primarily localized within the dermis and receive lower doses of UVB radiation than epidermal cells, they function as critical stromal amplifiers of tissue damage. In response to cytokines such as TNF-α and IL-1α released by irradiated keratinocytes, superficial dermal fibroblasts activate signaling circuits that trigger the secretion of specific chemokines, including CCL2 and CCL8. This chemokine gradient recruits MMP9+CD14+ myeloid cells, which serve as the primary source of matrix metalloproteinase-9 (MMP-9) within this circuit. The activity of these enzymes facilitates the degradation of structural components such as collagen IV, contributing to basement membrane disruption and the progression of UV-induced cutaneous inflammation [17].

### 4.5. Langerhans Cells

Langerhans cells, specialized epidermal dendritic cells, represent a critical component of cutaneous immune surveillance and are increasingly recognized as active regulators of UVB-induced skin responses. Following UVB exposure, Langerhans cells undergo functional activation and migration to draining lymph nodes, contributing to antigen presentation and immune modulation. Recent evidence further demonstrates that Langerhans cells orchestrate early innate immune responses by regulating the expression of neutrophil-attracting chemokines, including CXCL1 and CXCL2, thereby promoting rapid neutrophil recruitment to damaged skin. Recruited neutrophils subsequently release TNF-α, which facilitates the elimination of severely damaged keratinocytes through the formation of sunburn cells when DNA lesions exceed the cellular repair capacity. This Langerhans cell–neutrophil axis contributes to epidermal homeostasis by preventing the persistence of mutagenic cells and limiting the accumulation of genomic instability associated with photocarcinogenesis [15].

### 4.6. Mast Cells

Mast cells are long-lived resident immune cells predominantly located in the dermis, where they are strategically distributed around blood vessels, peripheral nerves, hair follicles, and glands, allowing them to rapidly respond to environmental stressors and tissue injury. These cells contain cytoplasmic granules rich in biologically active mediators, including histamine, proteases, cytokines, growth factors, and lipid mediators that can profoundly influence vascular, immune, and neural responses within the skin [12]. Recent evidence further highlights the contribution of mast cells to the altered cutaneous microenvironment and neurovascular interactions associated with chronic photodamage and pigmentary disorders, emphasizing their role beyond classical allergic responses [18].

UVB irradiation promotes mast cell activation and contributes to the development of local inflammatory responses in the skin. Following activation, mast cell-derived mediators increase vascular permeability, facilitate leukocyte recruitment, and amplify inflammatory signaling within UV-exposed tissues. In addition to their pro-inflammatory functions, dermal mast cells have been implicated in UV-induced immunomodulation through their migration to draining lymph nodes and the production of immunoregulatory mediators, including interleukin-10 (IL-10), which contribute to systemic immune suppression following UV exposure [12]. These functions place mast cells at the interface between inflammation, vascular responses, and neuroimmune signaling during UVB-induced skin damage [12,18].

## 5. Effects of UVB Radiation in the Skin

UVB radiation is primarily responsible for the inflammatory and deleterious effects associated with solar exposure. Over time, chronic UVB exposure contributes to photoaging, immunomodulation, DNA damage, and the development of skin cancer [19]. Nevertheless, solar radiation also exerts beneficial effects on the skin, including antibacterial activity and the production of active vitamin D from sterol precursors.

Two major mechanisms are involved in UV-induced tissue damage. First, DNA lesions may result directly from the absorption of mutagenic radiation, leading to the formation of pyrimidine dimers and other photoproducts. Second, DNA damage may occur indirectly through the interaction of UV radiation with biological chromophores, generating ROS that induce oxidative modifications capable of reaching the cell nucleus [20].

UVB radiation promotes oxidative stress through the excessive generation of ROS. Under physiological conditions, ROS are counterbalanced by endogenous antioxidant defense systems, including reduced glutathione (GSH) and CAT. However, excessive ROS production, particularly of superoxide anion (O_2_•^−^), overwhelms these antioxidant defenses and leads to their depletion. GSH directly neutralizes several ROS, participates in the detoxification of numerous compounds, and serves as an essential cofactor for antioxidant enzymes. CAT, a heme-containing antioxidant enzyme associated with peroxisomes, catalyzes the conversion of hydrogen peroxide (H_2_O_2_) into water and molecular oxygen [21,22].

Both O_2_•^−^ and H_2_O_2_ can damage a variety of cellular macromolecules. One of their most harmful effects is the induction of lipid peroxidation (LPO). Polyunsaturated fatty acids within cellular membranes are primary targets of ROS attack, resulting in loss of membrane integrity. Furthermore, increased production of O_2_•^−^ and H_2_O_2_ occurs in parallel with enhanced LPO. The products and by-products generated during this process are cytotoxic and progressively trigger oxidative damage and cellular apoptosis [6].

O_2_•^−^ also promotes leukocyte recruitment and activates inflammatory cytokine cascades. The inflammatory infiltrate, composed mainly of neutrophils and monocytes/macrophages, induces keratinocyte apoptosis, resulting in the formation of sunburn cells, the hallmark histopathological feature of UVB-induced skin damage. UVB-induced inflammation also manifests as erythema, edema, and epidermal thickening. These pathological changes are associated with leukocyte infiltration and increased production of pro-inflammatory cytokines, particularly TNF-α and interleukin-1 beta (IL-1β) [22]. This section presents the main mechanisms involved in the effects of UVB radiation on the skin. It will provide a concise and precise description of the experimental results, their interpretation, and the experimental conclusions that can be drawn.

### 5.1. Inflammatory and Oxidative Stress Pathways

**Figure 4:**
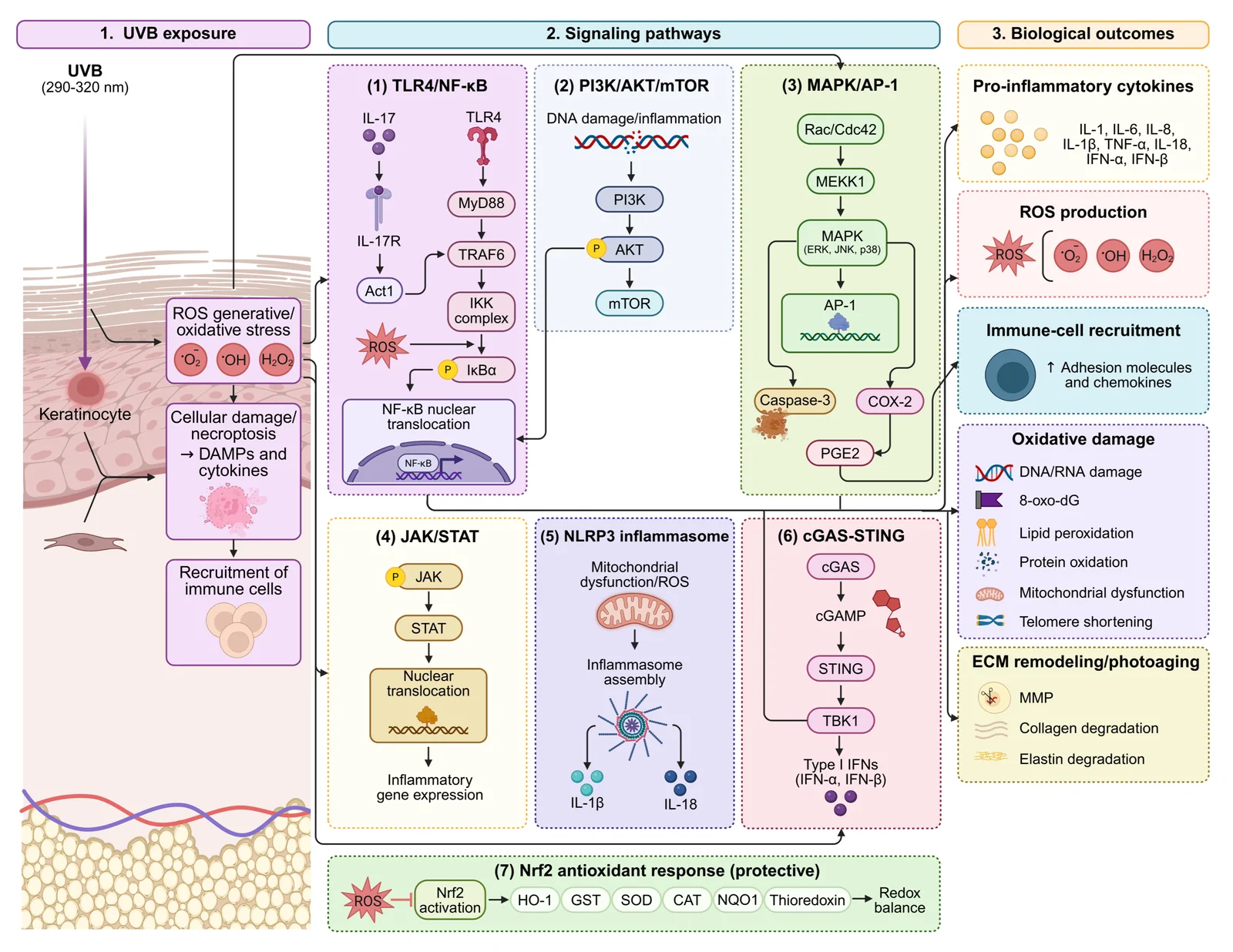
Main molecular mechanisms involved in UVB-induced skin inflammation and oxidative damage. UVB radiation (290–320 nm) acts predominantly on epidermal keratinocytes, promoting the generation of ROS, oxidative stress, cell damage, necroptosis, and the release of DAMPs and inflammatory mediators. These initial events activate interconnected signaling pathways, including the TLR4/MyD88/TRAF6/NF-κB, PI3K/AKT/mTOR, MAPK/AP-1, JAK/STAT, NLRP3 inflammasome, and cGAS–STING pathways. Activation of NF-κB, AP-1, and STATs promotes the expression of pro-inflammatory cytokines and chemokines, while MAPK signaling additionally induces COX-2/PGE2 and contributes to extracellular matrix remodeling. Mitochondrial dysfunction and the release of cytosolic DNA promote the activation of the NLRP3 inflammasome and the cGAS-STING complex, leading to the production of IL-1β, IL-18, and type I IFN. These mechanisms promote the recruitment of immune cells, the additional production of ROS, oxidative damage to cellular macromolecules, and MMP-mediated collagen and elastin degradation, thus contributing to cutaneous inflammation and photoaging. In parallel, the activation of the Nrf2 antioxidant response induces HO-1, GST, SOD, CAT, NQO1, and thioredoxin, providing a protective mechanism that limits ROS accumulation and contributes to redox homeostasis—a response that can be blocked by excess ROS. Abbreviations: Act1, NF-κB activator 1; AP-1, activator protein 1; CAT, catalase; cGAMP, cyclic GMP-AMP; cGAS, cyclic GMP-AMP synthase; COX-2, cyclooxygenase-2; DAMPs, damage-associated molecular patterns; GST, glutathione S-transferase; HO-1, heme oxygenase-1; IFN, interferon; IKK, IκB kinase; IL, interleukin; JAK, Janus kinase; JNK, N-terminal c-Jun kinase; MAPK, mitogen-activated protein kinase; MEKK1, mitogen-activated kinase kinase 1; MMP, matrix metalloproteinase; mTOR, mechanistic target of rapamycin; MyD88, myeloid differentiation response 88; NF-κB, nuclear factor kappa B; NLRP3, NLR family pyrin domain containing 3; NQO1, NAD(P)H quinone dehydrogenase 1; Nrf2, nuclear factor erythroid 2-related factor 2; PGE2, prostaglandin E2; PI3K, Phosphoinositide 3-kinase; ROS, reactive oxygen species; SOD, superoxide dismutase; STAT, signal transducer and activator of transcription; STING, interferon gene stimulator; TBK1, TANK-binding kinase 1; TLR4, Toll-like receptor 4; TNF-α, Tumor necrosis factor-alpha; TRAF6, TNF receptor-associated factor 6; UVB, ultraviolet B radiation. Created in BioRender. Verri, W.A. (2026) https://BioRender.com/v4q6fyt.

Figure 4 summarizes the main molecular mechanisms involved in UVB-induced skin inflammation and oxidative damage. Skin inflammation after exposure to UVB radiation is initiated by ROS, which accumulate after the depletion of enzymatic and non-enzymatic antioxidants. Inflammatory signaling pathways can also be activated by damage-associated molecular patterns (DAMPs), which are molecules derived from tissue injury [23,24]. Necroptotic cells can secrete DAMPs and pro-inflammatory cytokines to promote immune cell infiltration [25]. Depending on the level of exposure, activated keratinocytes and fibroblasts can produce pro-inflammatory signals and recruit immune cells, increasing the magnitude of inflammation [26].

The Toll-like receptor 4 (TLR4), a transmembrane protein, can activate the nuclear factor kappa B (NF-κB) signaling pathway via a myeloid differentiation factor 88 (MyD88)-dependent pathway. This pathway can activate tumor necrosis factor receptor-associated factor 6 (TRAF6), leading to activation of the IKK complex, which is responsible for IκBα phosphorylation and subsequent nuclear translocation of NF-κB. In HaCaT cells, TRAF6 can also be activated by the interleukin-17 (IL-17) signaling pathway, which binds to its IL-17R receptor and recruits the adaptor protein Act1 [27]. In addition, ROS can directly activate NF-κB through the phosphorylation of protein residues of IκBα. The NF-κB pathway, therefore, induces the expression of inflammatory cytokines, such as interleukin 1 (IL-1), interleukin 6 (IL-6), interleukin 8 (IL-8), IL-1β, and TNA-α [23,24,25,26,27,28].

The Phosphoinositide 3-kinase (PI3K)/AKT signaling pathway is activated in response to DNA damage and inflammation. AKT phosphorylation by PI3K regulates the proliferation of keratinocytes and NF-κB phosphorylation at the Ser536 residue. In this way, the PI3K/AKT/NF-κB pathway can lead to an excessive production of pro-inflammatory cytokines and intensify the production of ROS [29]. The inflammation generated can activate the AKT/mTOR/NF-κB pathway, where AKT acts directly by activating the mTOR complex [30].

In skin exposed to UVB radiation, the GTP-binding proteins Rac and Cdc42 are activated on the surface of fibroblast cell membranes. These proteins bind to MEKK1 and activate the mitogen-activated protein kinase (MAPK) pathway [31]. Downstream, activation of p38 proteins and N-terminal kinase c-Jun (JNK) leads to increased expression of cyclooxygenase-2 (COX-2), activating protein-1 (AP-1), and caspase-3 [32]. COX-2 regulates the production of prostaglandin 2 (PGE_2_), which is associated with immune cell infiltration [33].

Activation of the JAK-STAT signaling pathway plays a role in the subsequent intracellular signal transduction of most inflammatory cytokines [32]. ROS activates the phosphorylation of Janus tyrosine kinases (JAKs), which activate signal transducer and transcription-activating proteins (STATs). STATs are translocated to the nucleus to regulate different genes involved in the inflammatory process [34].

Another cutaneous inflammatory response is the activation of the NLR family pyrin domain containing protein 3 (NLRP3) inflammasome in keratinocytes, which consists of a family of proteins containing leucine-rich repeats that participate in the process of secretion of the interleukins IL-1β and IL-18 [35]. In addition to the inflammasome, activation of the cGAS-STING pathway by ROS or DNA damage has been documented in UVB-irradiated HaCaT cells. The role of mitochondria in activating the NLRP3 inflammasome through the leakage of mtDNA into the cytoplasm through disruption of mitochondrial fission/fusion dynamics is also discussed [36]. The presence of DNA in the cytoplasm leads to the activation of the cGAS (cytosolic DNA sensor), generating a chemical messenger called cGAMP. The STING transmembrane protein is activated in the endoplasmic reticulum by cGAMP binding. After that, STING forms a complex with TBK1, which activates transcription factors associated with the production of type I interferons (IFN) and inflammatory cytokines by the NF-κB pathway [37].

Acute and chronic exposure to UVB radiation induces both direct and indirect cellular damage through the generation of ROS. These highly reactive intermediates destabilize cellular components and trigger chain reactions that rapidly compromise biomolecular integrity, leading to telomere shortening, mitochondrial dysfunction, membrane degradation, and oxidative modification of structural and enzymatic proteins and the generation of LPO and macromolecular crosslinks, skin enzyme depletion, cytokine induction and the formation of 8-oxo-2′-deoxyguanosine. Activation of NF-κB is associated with UVB-induced oxidative modifications of cellular membrane components [38,39,40].

Oxidative stress activates MAPK pathways, including Extracellular Signal-Regulated Kinase (ERK), JNK, and p38. These pathways mediate cellular responses to stress, including inflammation, cell survival, and photoaging. Nrf2 drives the expression of a wide range of genes involved in antioxidant defense, including heme oxygenase-1 (HO-1), glutathione S-transferases (GSTs), superoxide dismutase (SOD), CAT, NAD(P)H quinone dehydrogenase 1 (NQO1), and thioredoxin. These enzymes work together to neutralize ROS, repair oxidative damage, and maintain cellular redox balance [41,42].

MAPKs trigger the transcription activity of the AP-1 protein, which enhances the role of different metalloproteinases (MMPs). AP-1 binds to promoter elements in the DNA that regulate the transcription of MMPs. MMPs are enzymes that degrade various extracellular matrix components, such as collagen and elastin. UV-induced activation of AP-1 leads to increased transcription of MMPs, which accelerates collagen breakdown in the skin, contributing to photoaging [36].

ROS comprise a heterogeneous group of highly reactive oxygen- and nitrogen-derived molecules that function in both cellular signaling and oxidative damage pathways. Under physiological conditions, ROS are generated through enzymatic and non-enzymatic processes, with mitochondria representing a major intracellular source. During oxidative phosphorylation, electron leakage from complexes I and III of the electron transport chain promotes the partial reduction of molecular oxygen, leading to superoxide formation. Additional ROS production arises from membrane-associated enzymes such as NADPH oxidases (NOX) and xanthine oxidase, which generate superoxide and H_2_O_2_ during redox reactions and purine metabolism, respectively [43,44].

In melanocytes, melanogenesis constitutes a major source of oxidative stress. Tyrosinase-mediated oxidation of tyrosine and 3,4-dihydroxy-L-phenylalanine (L-DOPA) generates reactive quinone intermediates and O_2_•^−^ throughout melanin biosynthesis. Subsequent redox cycling reactions further amplify ROS accumulation. Although tyrosinase-related protein 2 (TRP2) exerts a protective role by increasing intracellular glutathione levels and limiting quinone-mediated toxicity, excessive ROS production can overwhelm antioxidant defenses.

Physiologically, ROS participate in pathways regulating proliferation, differentiation, and immune responses. However, under conditions such as UVR exposure, inflammation, or tumorigenesis, ROS levels become markedly elevated, particularly in melanocytes due to their high melanogenic activity. This oxidative imbalance disrupts cellular homeostasis, promoting mitochondrial dysfunction, DNA damage, impaired cell viability, and ultimately, malignant transformation [45].

Melo et al. (2025) [46] described that the marked increase in O_2_•^−^ production observed in the UVB-stimulated group may be attributed not only to the high energy transfer capacity of UVB radiation, but also to the pronounced neutrophil infiltration within the tissue. Upon UV irradiation, neutrophils exhibit an intense oxidative burst response and release granule-derived components into the extracellular matrix, further exacerbating ROS accumulation and oxidative tissue damage.

### 5.2. Neuroimmune and Neuroendocrine Communication

**Figure 5:**
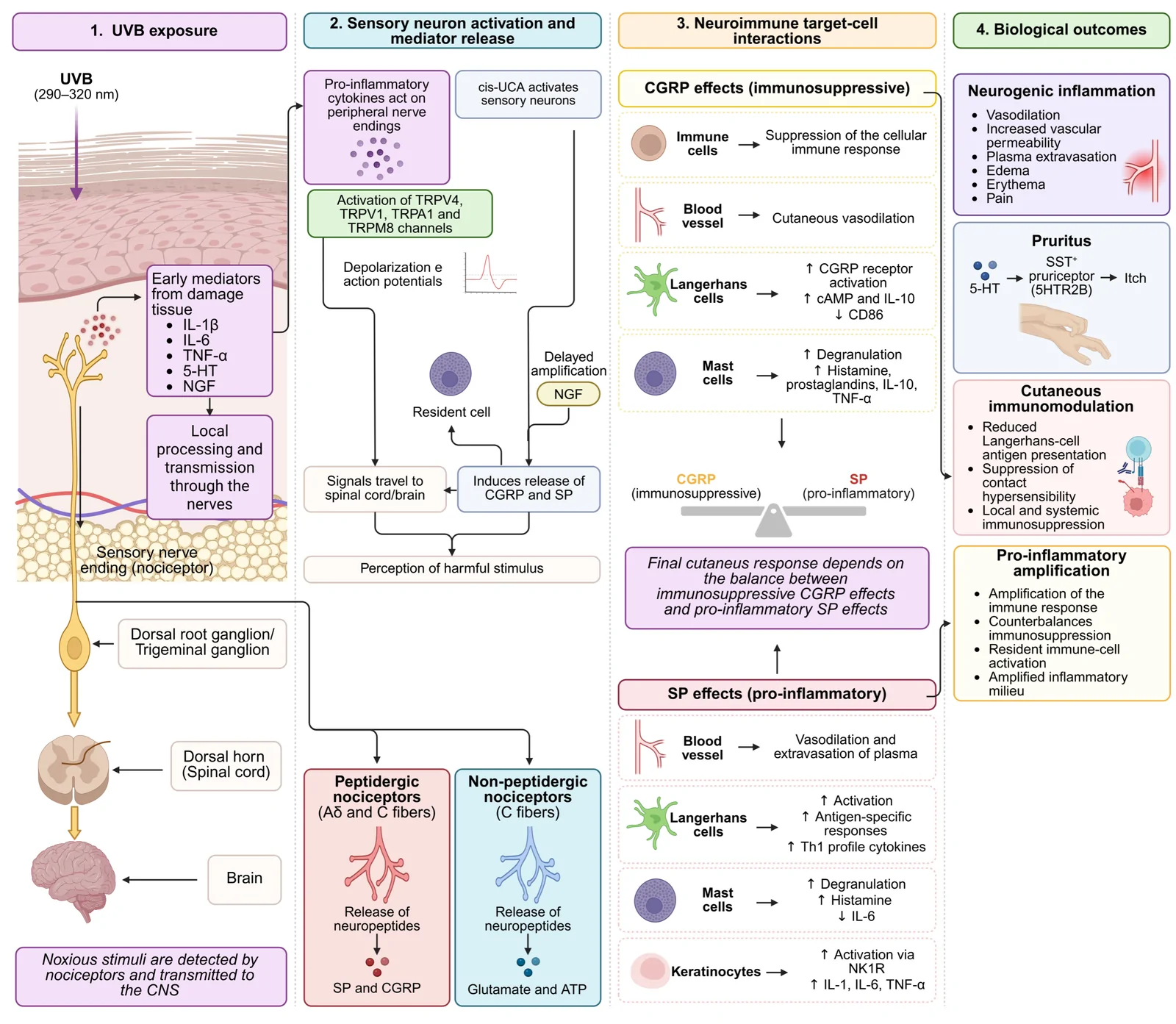
Neuroimmune mechanisms involved in the cutaneous response to UVB radiation. UVB radiation (290–320 nm) induces the release of inflammatory, photoinduced, and neuroendocrine mediators in the skin, including IL-1β, IL-6, TNF-α, serotonin (5-HT), nerve growth factor (NGF), and cis-urocanic acid (cis-UCA). These signals, along with the activation of transient receptor potential (TRP) channels, stimulate peripheral sensory nerve endings, promoting neuronal depolarization, the generation of action potentials, and the transmission of nociceptive signals through sensory ganglia and the spinal cord to the brain. Peptidergic nociceptors release SP and CGRP, while non-peptidergic nociceptors predominantly utilize glutamate and ATP. NGF produced after UVB exposure contributes to the late amplification and maintenance of neuroimmune signaling, stimulating the release of neuropeptides. CGRP and SP subsequently act on resident and immune cells, but exert partially opposing effects. CGRP predominantly favors immunomodulatory and immunosuppressive responses, promoting vasodilation, suppressing cellular immune responses, reducing antigen presentation by Langerhans cells through increased cAMP and IL-10 and decreased CD86, and modulating mast cell activity. In contrast, SP predominantly promotes inflammatory responses, inducing vasodilation and plasma extravasation, activating Langerhans cells, stimulating mast cell degranulation, and increasing the production of IL-1, IL-6, and TNF-α by keratinocytes via the NK1R receptor. Platelet-derived 5-HT additionally activates SST^+^ pruriceptors via the 5-HTR2B receptor, contributing to UVB-induced pruritus. Together, these interactions contribute to neurogenic inflammation, pruritus, cutaneous immunomodulation, and amplification of local inflammatory responses, with the final cutaneous outcome dependent, in part, on the balance between CGRP-mediated immunosuppressive effects and SP-mediated pro-inflammatory effects. Abbreviations: 5-HT, serotonin; 5-HTR2B, 5-hydroxytryptamine receptor 2B; ATP, adenosine triphosphate; cAMP, cyclic adenosine monophosphate; CGRP, calcitonin gene-related peptide; cis-UCA, cis-urocanic acid; CNS, central nervous system; IL, interleukin; NGF, nerve growth factor; NK1R, neurokinin-1 receptor; SP, substance P; SST, somatostatin; TNF-α, Tumor necrosis factor-alpha; TRP, transient receptor potential; TRPA1, transient receptor potential ankyrin 1; TRPM8, transient receptor potential melastatin 8; TRPV1, transient receptor potential vanilloid 1; TRPV4, transient receptor potential vanilloid 4; UVB, ultraviolet B radiation. Created in BioRender. Verri, W.A. (2026) https://BioRender.com/v4q6fyt.

The skin represents not only a passive physical barrier but also a highly specialized neuroimmune interface [13]. Figure 5 summarizes the main neuroimmune mechanisms involved in the cutaneous response to UVB radiation. Skin is innervated by primary sensory nerve endings responsible for detecting external stimuli, whether mechanical, thermal, or chemical, and distinguishing them in terms of intensity and harmfulness. These neurons have their cell bodies located in ganglia, such as the dorsal root ganglia (DRG) and the trigeminal ganglia, and project both to peripheral tissues and the dorsal horn of the spinal cord, thus linking the peripheral tissues to the central nervous system [14].

Among the classes of sensory neurons present in the skin are nociceptors, which are responsible for detecting noxious stimuli, leading to the development of pain and protective responses [14]. These can be classified into two subclasses: (1) peptidergic, responsible for the production and release of neuropeptides (for example, SP and CGRP); and (2) non-peptidergic, which primarily use glutamate and ATP for signal transmission [14,47].

In the presence of noxious stimuli, various mediators are released, such as the pro-inflammatory cytokines IL-1β, IL-6, and TNF-α, which are recognized by peripheral nerve endings present in sensory neurons [14]. This recognition leads to depolarization of the neuronal membrane and the generation of action potentials, which are integrated in the spinal cord and conducted to the brain for perception of the noxious stimulus [14].

Furthermore, nociceptors release neuropeptides and neurotransmitters into the skin, promoting local vasodilation and increased capillary permeability, as well as modulation of immune responses—important mechanisms for host defense and maintenance of tissue homeostasis [14]. Specifically, neuropeptides such as SP and CGRP act as central regulators of cutaneous immune responses through the activation of immune cells, vascular responses, tissue remodeling, and counter-regulation of excessive inflammation, actions that highlight the skin as an active neuroimmune interface [13]. However, neuroimmune communication induced by UVR does not depend only on the release of neuropeptides but also involves the production of neuroendocrine mediators by resident skin cells, especially keratinocytes. After exposure to UVB radiation, keratinocytes can produce molecules such as nerve growth factor (NGF). These mediators act in an integrated manner on sensory neurons, melanocytes, keratinocytes, and other immune cells, contributing to cutaneous immunomodulation and representing a bridge between epidermal damage caused by UVB and the activation of neuroimmune circuits responsible for regulating inflammation, antigen presentation, and local or systemic immunosuppression [48].

A complete and detailed review by Slominski et al. (2024) integrates evidence that UV radiation regulates body homeostasis through neural, immune, and endocrine signals generated primarily in the skin [49]. The authors proposed the concept of photo-neuro-immuno-endocrinology to describe how UVR regulates communication among the skin, nervous system, immune system, and endocrine axis. That is, cutaneous exposure to UV radiation induces chemical, hormonal, immunological, and neuronal signals that are processed locally in the skin and transmitted by cutaneous nerves or by humoral mediators released into the circulation. In this context, peripheral sensory neurons participate as important communication pathways between irradiated skin and systemic regulatory centers, since the mediators induced by UVR can activate or be released by cutaneous nerve fibers. This neuroimmune network contributes to the modulation of inflammation, immunosuppression, and the activation of neuroendocrine axes, reinforcing the concept that the skin acts as a sensory and systemic regulatory organ after exposure to UVR [49].

Several studies highlight that the neuroimmune system, mainly through interactions between neuropeptides and cells, plays an important role in the mechanisms and effects caused by UVR. Experimental studies have demonstrated increased SP immunoreactivity in the skin after exposure to UVR, suggesting activation of sensory nerve fibers and amplification of cutaneous neuroimmune communication. This finding reinforces that the response to UVB radiation involves not only keratinocytes and resident immune cells but also peripheral neuronal circuits capable of modulating local inflammation [48].

Furthermore, it has been shown that cis-urocanic acid (cis-UCA), an agent with potential anti-inflammatory and antiproliferative activity formed in the skin through the isomerization of trans-urocanic acid (trans-UCA) by UVB radiation, is responsible for activating peripheral sensory neurons and inducing the release of CGRP and SP [48,50]. This mechanism suggests that photochemical products generated in the epidermis can transform an environmental stimulus, in this case, UVB radiation, into neuroimmune signals capable of altering the inflammatory and immunological responses of the skin, as well as indirectly contributing to the activation of immune cells [48].

However, these neuropeptides appear to exert distinct effects on the cutaneous immune response. While CGRP is strongly associated with UVR-induced immunosuppression, namely, excessive exposure to UVR can inhibit the immune response and impair the development of contact hypersensitivity when skin exposure to a new substance occurs [51], SP has a more pro-inflammatory profile and may favor local immune responses. This paradox suggests that the final effect of neuronal activation induced by UVB radiation depends on the balance between immunosuppressive and pro-inflammatory neuropeptides released into the cutaneous microenvironment [48].

Garssen et al. (1998) demonstrated precisely these distinct mechanisms among neuropeptides in the development of UVB radiation-induced immunosuppression [52]. Using a murine model of contact hypersensitivity to picryl chloride, the authors observed that exposure to UVB radiation systemically suppressed the cellular immune response. This suppression was mainly associated with the participation of CGRP, since blocking the signaling of this neuropeptide reduced UVB radiation-induced immunosuppression. In contrast, tachykinins, including SP, did not play an essential role in this model [52].

In the chronology of the inflammatory response, CGRP participates in the initial vascular changes associated with UVR exposure. Studies in experimental models have demonstrated that, after exposure to UVB radiation, sensory neurons release CGRP, which directly contributes to cutaneous vasodilation, suggesting its participation in the initial phase of neurogenic inflammation [48,53]. Similar effects have been observed for neuronal nitric oxide (NO). Interestingly, CGRP has been shown to act independently of endothelial NO to induce vasodilation [53]. Unlike CGRP, SP is classically associated with the acute inflammatory response to UVB radiation, since, after exposure to UVR, this neuropeptide participates in the initial vascular changes in the skin, including vasodilation and plasma extravasation, directly contributing to the formation of edema and erythema and, consequently, increased neurogenic inflammation [48].

One neurotransmitter involved in the initial phase of the inflammatory response triggered by UVB radiation is serotonin (5-HT) [54]. Recent studies have demonstrated a novel platelet-neuron axis, in which platelet-derived 5-HT directly activates SST^+^ pruriceptors, specialized sensory nerve endings in the skin responsible for detecting stimuli that generate the sensation of itching, through 5-HT receptor 2B to mediate UVB radiation-induced itching—findings that further expand our understanding of cutaneous neuroimmune communication [54].

Furthermore, neuropeptides also play a role in subsequent mechanisms of immune regulation. Langerhans cells are one of the main immunological targets of CGRP, given the close anatomical association between peptidergic nociceptors and these cells, suggesting direct communication between the sensory nervous system and the antigen-presenting cells of the epidermis [41,48]. Mechanistically, CGRP reduces the ability of Langerhans cells to present antigens and stimulate T lymphocytes by activating the CGRP receptor, increasing cyclic adenosine monophosphate (cAMP), and inducing IL-10 [48], resulting in a reduction in co-stimulatory molecules (e.g., CD86) and, consequently, reduced antigen presentation [48,55].

These findings are supported by studies showing that topical application of CGRP mimicked several effects of UVR, including the reduction of Langerhans cells and the suppression of contact hypersensitivity [48,53]. Furthermore, blocking CGRP with an antagonist reduced UVR-induced immunomodulation, indicating that this neuropeptide is a functional mediator of cutaneous immunosuppression after UVB radiation exposure [48,53]. Similar effects were observed by Kitazawa and Streilein (2000), who reported that CGRP plays a central role in cutaneous immunological tolerance by promoting an IL-10- and dendritic cell-dependent tolerogenic environment, and that this mechanism contributes to UVB radiation-induced immunosuppression [56].

Similarly, repeated or chronic exposure to UVR increases the neuropeptide content of epidermal nerve fibers and, consequently, increases cutaneous levels of SP and CGRP [57]. This increase correlates with a reduction in the contact hypersensitivity response and suggests that chronic exposure to UVR may remodel the cutaneous neuroimmune axis, favoring an immunosuppressive microenvironment mediated, in part, by CGRP [48]. Conversely, SP can activate Langerhans cells, favoring the initiation of antigen-specific responses and the production of cytokines associated with a Th1 profile, suggesting that this neuropeptide may act as a mediator of amplification of the cutaneous immune response [48,58].

Together with Langerhans cells, dermal mast cells represent an important target of neuropeptides released after exposure to UV radiation [56]. CGRP can induce mast cell degranulation, promoting the release of mediators such as histamine, prostaglandins, IL-10, and TNF-α, which can act locally on keratinocytes and Langerhans cells and contribute to suppression of the cutaneous immune response and impairment of delayed hypersensitivity development [59,60,61,62,63]. NO, released along with CGRP by sensory neurons, also appears to participate in this process, although its mechanism is not yet fully understood [60].

In the case of SP, mast cells are also induced to release histamine, contributing to vascular and inflammatory responses. However, mast cell activation by SP does not completely reproduce the classic activation mediated by the high-affinity receptor for immunoglobulin E (FcεRI), since it can occur without robust induction of TNF-α or IL-8, and in some contexts, there is a reduction in IL-6 production [48].

Another target cell of neuropeptides is keratinocytes. It is currently known that SP can act directly on keratinocytes through the neurokinin-1 receptor (NK1R), stimulating the production of pro-inflammatory cytokines such as IL-1, IL-6, and TNF-α and creating an epidermal microenvironment favorable to inflammation. Thus, SP contributes to transforming UVB-induced neural activation into a local keratinocyte-mediated inflammatory response [13,45,52,61]. All these findings indicate that UVB radiation modulates cutaneous and systemic immunity through functional communication among photoinduced molecules, sensory neurons, immune and resident cells, and inflammatory mediators.

As observed so far, unlike CGRP, SP is not predominantly described as a mediator of UVR-induced immunosuppression. On the contrary, in some experimental models, SP activation favored cutaneous immunity and was able to reverse the local suppression of contact hypersensitivity induced by UVB radiation, indicating that SP can counterbalance, in certain contexts, the immunosuppressive effects associated with radiation exposure [64]. This difference suggests that neuropeptides released by the same neurons may exert distinct and even opposing functions in the cutaneous response to UVB radiation, and that the final immunological outcome after UVR exposure may depend on the balance between these neuropeptides in target cells and vascular responses.

However, the biological activity of SP in skin exposed to UVB radiation depends not only on its release by nociceptors but also on its enzymatic degradation. SP is more sensitive than CGRP to the action of neuropeptide-degrading enzymes, such as neutral endopeptidase (NEP), angiotensin-converting enzyme (ACE), and dipeptidyl peptidase 4 (DPP-4), and the duration and intensity of its effects can be regulated after UVB exposure [64].

NGF is a neuroimmune mediator produced mainly by keratinocytes exposed to UVR and can amplify and sustain communication between the epidermis and sensory neurons [64]. Following irradiation, keratinocytes increase NGF production, which can act on sensory neurons, promoting the release of neuropeptides such as CGRP and SP, which subsequently act on resident skin cells, such as mast cells. Thus, it can be said that NGF occupies an intermediate position in neuroimmune communication, as it connects irradiated keratinocytes to neuronal activation and the subsequent modulation of mast cells and immune cells [48,49].

Furthermore, NGF production by keratinocytes has been observed to peak between 8 and 12 h after exposure to UVB radiation [53,65], indicating that this neuroendocrine hormone does not represent an immediate response to damage, but rather a delayed response to radiation that is responsible for sustaining neuroimmune activation in the subsequent hours. That is, the initial release of neuropeptides occurs due to the production of cis-UCA or other factors present in the inflammatory microenvironment and is sustained for hours after the initial stimulus through the delayed production and release of NGF [51].

Transient receptor potential (TRP) channels constitute a family of ion channels involved in the detection and transduction of different physical and chemical stimuli, including temperature, mechanical stress, chemical irritants, and inflammatory mediators. In the skin, these channels are expressed not only in sensory neurons but also in non-neuronal cells, such as keratinocytes and immune cells, indicating that the epidermis actively participates in the perception of and response to environmental stimuli [66].

Mechanistically, UVB radiation activates TRP vanilloid 4 (TRPV4) channels in epidermal keratinocytes, promoting calcium (Ca^2+^) influx, increased endothelin-1 (ET-1) expression, and the development of radiation-associated pain and skin damage [66]. In mice with keratinocyte-specific TRPV4 deletion, UVB radiation exposure resulted in reduced mechanical and thermal hypersensitivity, decreased epidermal damage, and reduced recruitment of inflammatory cells. Furthermore, topical inhibition of TRPV4 reduced UVB radiation-induced pain, tissue damage, and ET-1 expression, indicating that keratinocytes function as active sensors of UVB radiation and can modulate cutaneous neuroimmune communication through signals such as ET-1 [67].

Similar findings were observed with respect to the TRP vanilloid 1 (TRPV1) channel. Lee et al. (2009) demonstrated that UVR activates TRPV1 channels in human keratinocytes, inducing persistent Ca^2+^ influx and contributing to matrix metalloproteinase-1 (MMP-1) expression [68]. Pharmacological blockade of TRPV1 with capsazepine or ruthenium red, as well as TRPV1 knockdown, reduced both Ca^2+^ influx and UVR-induced MMP-1 expression. These findings indicate that TRPV1 channels function as mediators of the keratinocyte response to UVR, connecting the activation of sensory ion channels to extracellular matrix remodeling [68].

Subsequent findings confirmed that TRPV1 functionally participates in the cutaneous response to UVR, possibly contributing to inflammatory and extracellular matrix remodeling events associated with photoaging [69]. In an in vivo model, pharmacological blockade of TRPV1 with 5′-iodoresiniferatoxin (I-RTX) reduced UVR-induced skin responses by inhibiting skin thickening and reducing the expression of MMP mRNA, pro-inflammatory cytokines such as IL-1β, interleukin 2 (IL-2), interleukin 4 (IL-4), and TNF-α, as well as COX-2 and p53 [69].

Furthermore, studies have shown that topical treatment with capsaicin reduced the suppression of the Mantoux response, a delayed hypersensitivity reaction to purified tuberculin protein derivative, induced by UVR in humans [70]. Since capsaicin acts primarily by activating TRPV1^+^ sensory neurons, these findings suggest that capsaicin-sensitive neuronal pathways participate in UVR-induced cutaneous immunomodulation. However, the study did not directly assess TRPV1 expression or specifically block TRPV1 channels; therefore, the involvement of this receptor should be interpreted as inferred from capsaicin use.

In addition, the participation of TRP ankyrin 1 (TRPA1) channels in UVB radiation-induced skin inflammation has also been demonstrated [71]. Single or repeated exposure to UVB radiation induces edema formation, inflammatory cell infiltration, histological changes, oxidative stress, epidermal hyperplasia, and increased TRPA1 expression. Topical blockade of TRPA1 with HC-030031 reduced these parameters, indicating that this channel contributes to inflammatory, oxidative, and proliferative responses associated with UVB radiation. Furthermore, experiments using resiniferatoxin-induced denervation suggested that both neuronal and non-neuronal TRPA1 participate in this process, although cutaneous nerve fibers appear to make a greater contribution to the full development of UVB radiation-induced inflammation [71].

Finally, activation of TRP melastatin 8 (TRPM8) channels reduces UVB radiation-induced PGE_2_ production in keratinocytes [72]. UVB radiation significantly increases PGE_2_ release, whereas TRPM8 activation, either by agonists such as menthol or by low temperatures, reduces this release. In this case, unlike the channels mentioned above, TRPM8 appears to play a regulatory and/or anti-inflammatory role in the cutaneous response to UVB radiation, partially counteracting the pro-inflammatory pathways mediated by TRPV1, TRPA1, and TRPV4 channels [72].

Together, these findings demonstrate that TRP channels represent fundamental components of neuroimmune communication induced by UVB radiation in the skin. When activated in keratinocytes, sensory neurons, and possibly immune cells, these channels convert the physical stimulus of radiation into cellular responses associated with Ca^2+^ influx, production of inflammatory mediators, neuronal activation, pain, tissue remodeling, and immunomodulation.

Furthermore, these findings demonstrate that UVB radiation activates a complex neuroimmune communication network in the skin, in which resident cells, sensory neurons, immune cells, and inflammatory mediators act in an integrated manner. Exposure to UVB radiation can generate photoinduced molecules, such as cis-UCA; stimulate the production of neuroendocrine hormones, such as NGF; activate ion channels, such as TRP channels; and induce the release of neuropeptides, such as CGRP and SP. These events contribute to the formation of an integrated neuroimmune communication network. Therefore, skin exposed to UVB radiation should be understood not only as a passive target of radiation but also as an active sensory and immunoregulatory organ.

## 6. Potential Therapeutic Strategies Targeting UVB-Induced Skin Damage

The elucidation of the molecular mechanisms underlying UVB-induced skin damage has enabled the development of targeted therapeutic and preventive strategies. These approaches aim to attenuate oxidative stress, modulate inflammatory signaling, preserve dermal matrix integrity, and regulate neuroimmune interactions. Mechanism-based interventions can be broadly categorized into antioxidant, anti-inflammatory, photoprotective, and neuroimmune-targeted strategies, which often act synergistically to confer comprehensive cutaneous protection (Table 1).

### 6.1. Antioxidant-Based Strategies

The excessive production of ROS following UVB exposure is a central and early event in skin damage, leading to LPO, protein oxidation, and DNA lesions [20]. ROS activate redox-sensitive transcription factors including NF-κB and AP-1, amplifying downstream inflammatory cascades. Antioxidant strategies therefore aim not only to neutralize ROS directly, but also to restore redox homeostasis and interrupt the oxidative-inflammatory axis that drives cutaneous pathology.

Plant-derived antioxidants have demonstrated protective effects in experimental models through multitarget mechanisms, addressing the ROS overload, inflammatory mediator upregulation, and structural degradation that characterize UVB-induced skin damage.

Classical antioxidants—including vitamins C and E, carotenoids, and polyphenols—have demonstrated protective potential against UVB-induced oxidative damage and inflammation in experimental and translational studies [92]. In addition to these classical antioxidants, studies have demonstrated that natural products and phytochemicals significantly attenuate oxidative stress markers and improve skin histopathological parameters after UVB exposure.

For instance, treatment with Cordia verbenacea extracts, administered both orally and topically, reduced LPO and inflammatory damage in UVB-irradiated skin of hairless mouse [73,74]. Similarly, *Nectandra cuspidata*-derived polyphenols protected fibroblasts and skin tissue by attenuating ROS generation and inflammatory mediators [93]. Additionally, quercetin, a flavonoid with established antioxidant and anti-inflammatory properties, reduces oxidative biomarkers and inflammatory infiltration in a mouse model, with microencapsulation markedly improving its bioavailability and efficacy [75].

Marine-derived compounds further expand this antioxidant arsenal. Dieckol, a phlorotannin isolated from Ecklonia cava, suppresses UVB-induced ROS accumulation, inhibits collagenase activity, promotes collagen synthesis, and downregulates pro-inflammatory cytokines through coordinated modulation of NF-κB, AP-1, and MAPK pathways in human dermal fibroblasts [76]. Similarly, phenolic acids derived from Panax ginseng preserve epidermal barrier function, reduce transepidermal water loss (TEWL), inhibit matrix MMPs, and suppress COX-2 and cytokine expression in a hairless mouse skin photodamage model, thus combining antioxidant, anti-inflammatory, and anti-photoaging effects [77].

A mechanistically compelling antioxidant strategy involves activation of the nuclear factor erythroid 2-related factor 2 (Nrf2) signaling pathway. Under basal conditions, Nrf2 is sequestered in the cytoplasm by Kelch-like ECH-associated protein 1 (Keap1); however, UVB-induced oxidative stress leads to Keap1 oxidation and Nrf2 nuclear translocation, upregulating antioxidant response element (ARE)-driven genes. Paradoxically, chronic UVB exposure can impair this endogenous defense, rendering pharmacological Nrf2 activation as a potential therapeutic strategy.

Phytochemicals such as sulforaphane, curcumin, and various flavonoids induce Nrf2 nuclear translocation, thereby upregulating endogenous cytoprotective enzymes including HO-1, SOD, catalase (CAT), and glutathione-S-transferase [78,79]. Critically, this strategy amplifies endogenous antioxidant capacity rather than relying solely on exogenous ROS scavenging—a distinction with important therapeutic implications, since it restores the cell’s own defense systems impaired by repeated UV exposure.

A major translational limitation of natural antioxidants is poor physicochemical stability and insufficient skin penetration when applied topically. Advanced delivery technologies have been developed to overcome these barriers, enhancing both bioavailability and the depth of antioxidant protection.

Microencapsulation strategies, such as quercetin-loaded microcapsule systems, enhance stability, control release kinetics, and improve dermal penetration, resulting in superior protection against UVB-induced oxidative and inflammatory damage [75]. Nanostructured lipid carriers and hydrogel systems similarly optimize skin delivery and prolong therapeutic effects [80]. Conventional nanocarriers such as liposomes, solid lipid nanoparticles, and nanoemulsions improve the stability and skin penetration of labile antioxidants such as vitamin C, enabling controlled and sustained release [94].

Plant-derived exosome-like nanoparticles represent an innovative, biocompatible platform with potential application in topical antioxidant therapy. Encapsulation of chebulinic acid in mulberry-derived nanoparticles improved dermal penetration and modulated IL-6 and IFN-β expression through MAPK signaling pathways [81]. Bioinspired synthetic systems such as polydopamine nanoparticles, which structurally mimic the melanin-like antioxidant system of skin cells, exhibit potent ROS-scavenging and anti-inflammatory properties [82], highlighting the convergence of natural and synthetic strategies in next-generation photoprotection.

Despite these promising findings, most antioxidant delivery systems and pharmacological approaches described above remain at the preclinical stage and further clinical studies are required to establish their safety, efficacy, optimal dosing and applicability in human photoprotection.

### 6.2. Anti-Inflammatory and Pro-Resolving Approaches

As discussed in Section 5.1, UVB-induced oxidative stress activates NF-κB and AP-1 transcription factors alongside MAPK cascades, driving increased production of TNF-α, IL-1β, IL-6, COX-2, and PGE_2_. Anti-inflammatory strategies targeting these pathways are therefore directly informed by the pathophysiology of UVB-induced cutaneous injury, and their therapeutic rationale is firmly grounded in the molecular events described throughout this review.

Natural compounds modulate UVB-induced inflammation at multiple levels, targeting both upstream kinase cascades and downstream inflammatory mediators. Polyphenols and flavonoids—including resveratrol, quercetin, hesperidin methyl chalcone, trans-chalcone, and naringenin—inhibit NF-κB activation, suppress cytokine production, and attenuate oxidative stress [75,78,79,83,84,85]. Their capacity to simultaneously target multiple inflammatory nodes reflects the pleiotropic nature of the UVB-induced signaling network.

COX-2 inhibition and downstream cytokine suppression represent particularly important anti-inflammatory mechanisms. Ginseng phenolic acids reduce IL-1β and IL-6 expression in UVB-exposed skin through this pathway [77].

Topical formulations incorporating Pimenta pseudocaryophyllus extract [95], hesperidin methyl chalcone [84], trans-chalcone [85], and naringenin [78] have demonstrated significant reductions in skin inflammation and oxidative markers in hairless mouse models, underscoring the translational value of optimized formulation strategies.

A significant conceptual and therapeutic advance in UVB research is the recognition that the resolution of inflammation is not a passive process, but an active, regulated program mediated by specialized pro-resolving lipid mediators (SPMs). Whereas classical anti-inflammatory strategies suppress inflammatory mediator production, SPMs actively orchestrate the clearance of inflammatory cells and restore tissue homeostasis—a distinction with profound pathophysiological relevance. Their main classes of compounds are: lipoxins, maresins, resolvins and protectins.

Lipoxin A4 inhibits UV-induced skin inflammation and oxidative stress via the ALX/FPR2 receptor pathway [86], and Maresin 1 reduces inflammatory and oxidative parameters following UVB exposure [3]. These mediators act on receptors expressed by keratinocytes, immune cells, and endothelium, integrating anti-inflammatory and reparative signals within the skin microenvironment. Resolvin D2 reduces oxidative stress, cytokine production, and NF-κB activation in UVB-irradiated hairless mouse skin [19]. Aspirin-triggered Resolvin D1 (AT-RvD1) attenuates leukocyte recruitment, oxidative damage, and inflammatory signaling pathways on UVB-induced inflammation and oxidative stress in hairless mice [46].

Acetylsalicylic acid exerts protective effects against UVB-induced skin pathology partially through pro-resolving mechanisms: its efficacy is reduced in the presence of the ALX/FPR2 receptor antagonist BOC-2, indicating dependence on lipoxin and resolvin signaling pathways [87].

These findings bridge classical pharmacology with the emerging biology of inflammation resolution, reinforcing the therapeutic relevance of targeting pro-resolving pathways in UVB-injured skin.

### 6.3. Photoprotective Strategies and Skin Barrier Preservation

Photoprotection remains the primary preventive strategy against UVB-induced damage. However, as the molecular mechanisms described earlier in this review demonstrate, conventional UV filters alone are insufficient to prevent the full spectrum of oxidative, inflammatory, and genotoxic injury triggered by UVB exposure [88]. This has driven interest in multifunctional agents that integrate physical UV attenuation with pharmacological activity targeting the downstream pathophysiological consequences of UV irradiation.

Botanical compounds such as quercetin, dieckol, and ginseng phenolics exhibit combined photoprotective activity that extends beyond UV absorption. Their effects encompass ROS scavenging, anti-inflammatory signaling, MMP inhibition, and restoration of epidermal barrier function [75,76,77], directly counteracting the oxidative-inflammatory-matrix degradation axis that underlies both acute UVB damage and chronic photoaging.

### 6.4. DNA Repair and Anti-Photoaging Approaches

Given that cyclobutane pyrimidine dimer (CPD) formation and 6-4 photoproduct accumulation are primary genotoxic events of UVB irradiation, topical formulations incorporating DNA repair enzymes represent a mechanistically rational preventive strategy. Photolyases and endonucleases have demonstrated efficacy in reducing UV-induced mutagenic photoproducts and cellular dysfunction [88], directly addressing the upstream genotoxic consequences of UVB exposure and complementing antioxidant and anti-inflammatory approaches.

Inhibition of MMPs is critical for preventing collagen degradation and the photoaging phenotype driven by chronic UVB exposure. UVB activates MMP-1 and MMP-3 through ROS-mediated AP-1 signaling, leading to collagen fragmentation. Both marine-derived (dieckol) and plant-derived (ginseng phenolic acids) compounds reduce MMP expression and promote collagen biosynthesis in UVB-exposed skin models [76,77], offering mechanistically grounded anti-photoaging protection.

### 6.5. Neuroimmune-Modulating Approaches

UVB exposure activates cutaneous sensory nerve endings, triggering the release of neuropeptides that modulate local inflammation, immune responses, and pain perception [96]. This neuroimmune axis—involving CGRP, SP, NGF, α-MSH, and endogenous opioids—constitutes a pathophysiologically relevant and pharmacologically targetable component of the UVB response. Therapeutic strategies directed at these mediators represent an emerging and predominantly experimental field in dermatological photoprotection. Although modulation of CGRP, SP, NGF and other neuroimmune pathways has shown promising effects in preclinical models, these approaches have not yet been established as standard clinical strategies.

### 6.6. Neuropeptide and Receptor-Based Strategies

CGRP antagonists delivered via microneedle systems reduce neurogenic inflammation and pain with minimal systemic effects [89], directly counteracting the neuropeptide-driven inflammatory amplification. TRPV1 channel modulation decreases neuropeptide release and macrophage activation [90], interrupting the sensory-immune crosstalk that sustains post-UVB neurogenic inflammation. β-endorphin signaling protects barrier function by inhibiting NF-κB and mTOR pathways [30]. Furthermore, the endocannabinoid system participates in the cutaneous response to UVB, as CB1/CB2 receptors are directly activated by UV irradiation and their signaling modulates NF-κB activation and MAPK cascades, thereby contributing to UVB-induced inflammation and representing a potential therapeutic target [91].

Moreover, UVB-induced expansion of proenkephalin^+^ regulatory T cells contributes to tissue repair and immune homeostasis [97], and urocortin 2 expression by dendritic cells mediates regulatory T cell-dependent immunosuppression following UV exposure [98]. Collectively, these findings establish that neuroimmune crosstalk is not merely a consequence of UVB injury but an active regulatory network with significant therapeutic potential.

### 6.7. Integrated Therapeutic Perspectives

The pathophysiological mechanisms of UVB-induced skin damage described throughout this review form an interconnected network in which oxidative stress triggers inflammatory signaling, inflammation activates neuroimmune pathways, and neuropeptides further modulate immune and oxidative responses. This biological complexity underscores the limitations of single-target approaches and supports the development of integrated, multi-pathway interventions.

Combination strategies integrating antioxidants, anti-inflammatory and pro-resolving mediators, neuroimmune modulators, and advanced drug delivery systems are likely to provide superior and more durable photoprotection relative to any individual approach. Advanced delivery platforms—including microencapsulation, nanocarriers, and nanoparticles—not only improve the bioavailability and skin penetration of individual bioactives but can enable simultaneous delivery of multiple compounds targeting distinct pathophysiological nodes.

## 7. Conclusions

**Figure 6:**
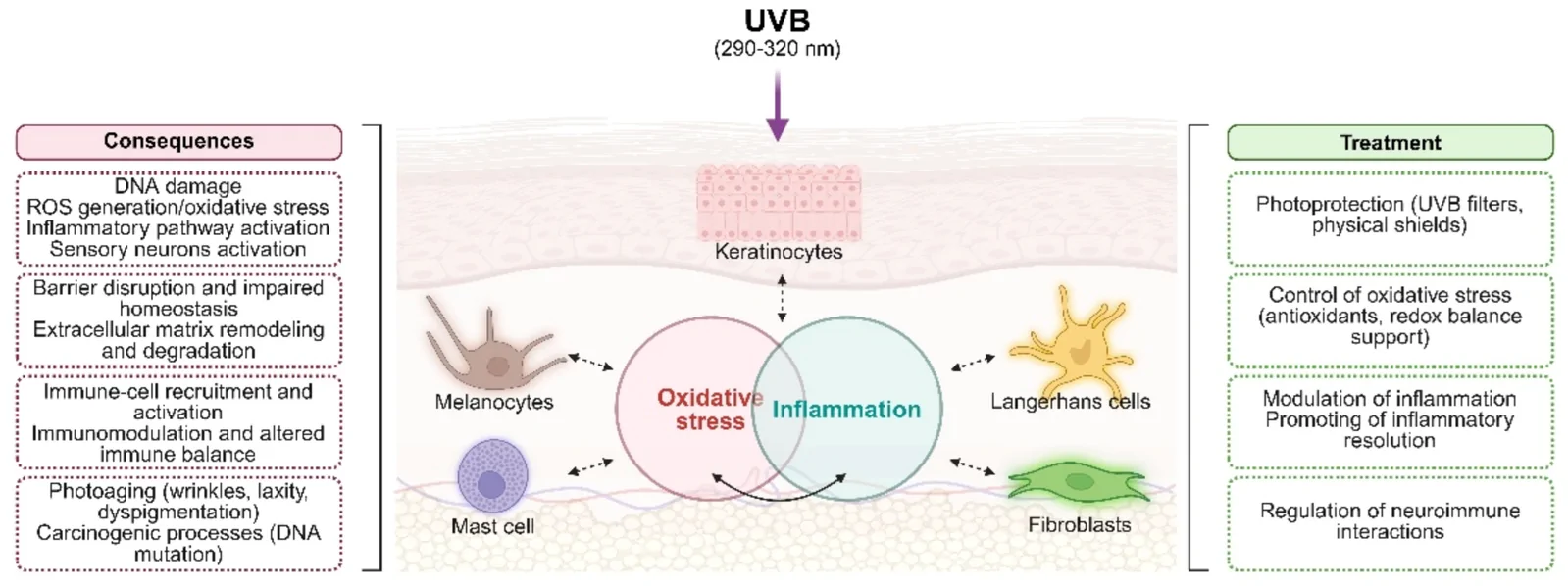
Integrated overview of UVB-induced skin damage and potential therapeutic strategies. UVB radiation (290–320 nm) primarily affects the epidermis, where keratinocytes represent the main cellular target, but the resulting response involves coordinated communication between melanocytes, Langerhans cells, mast cells, fibroblasts, and other resident and recruited cells. UVB exposure induces DNA damage, ROS generation and oxidative stress, activation of inflammatory pathways, and stimulation of sensory and neuroimmune mechanisms. Oxidative stress and inflammation form interconnected processes that amplify tissue injury and propagate responses through different skin compartments. These mechanisms contribute to the disruption of epidermal barrier function and tissue homeostasis, remodeling and degradation of the extracellular matrix, recruitment and activation of immune cells, altered immune regulation, photoaging, and carcinogenic processes. Since UVB-induced skin damage results from multiple interconnected pathways, potential preventive and therapeutic approaches include photoprotection, control of oxidative stress, modulation and resolution of inflammation, and regulation of neuroimmune interactions. Together, these strategies illustrate the importance of targeting multiple components of the skin response to limit UVB-induced damage and promote the restoration of tissue homeostasis. Abbreviations: ROS, reactive oxygen species; UVB, ultraviolet B radiation. Created in BioRender. Verri, W.A. (2026) https://BioRender.com/v4q6fyt.

Exposure to UVB radiation triggers a complex cutaneous response involving multiple cell types and interconnected molecular mechanisms (Figure 6). Although its penetration is largely confined to the epidermis, the absorption of UVB by cellular components initiates events that can propagate across different skin compartments. Damage to genetic material, increased production of ROS, and activation of inflammatory pathways are central events in this process and contribute to both acute responses and alterations associated with repeated or chronic exposure.

Oxidative stress and inflammation are closely interconnected in the cutaneous response to UVB radiation. Increased generation of ROS can damage lipids, proteins, DNA, and other cellular structures while simultaneously activating signaling pathways responsible for the production of inflammatory mediators. These mediators, in turn, promote the recruitment and activation of immune cells, thereby amplifying the tissue response. Depending on the intensity and duration of exposure, these mechanisms may contribute to disruption of skin barrier integrity, extracellular matrix remodeling, immunomodulation, photoaging, and the development of carcinogenic processes.

Beyond these classical mechanisms, recent evidence indicates that the cutaneous response to UVB also depends on extensive communication among resident cells, immune cells, and peripheral nerve endings. The production of photoinduced and neuroendocrine mediators, activation of sensory channels, and release of neuropeptides demonstrate that the skin does not function merely as a passive target of radiation, but rather as a sensory and immunoregulatory organ capable of integrating environmental stimuli with local and systemic responses. The involvement of keratinocytes, melanocytes, fibroblasts, Langerhans cells, mast cells, and sensory neurons further highlights the multicellular and dynamic nature of this response.

An integrated understanding of these processes broadens the possibilities for preventing and treating UVB-induced skin damage. Strategies directed toward a single mechanism may be insufficient given the complexity of the cutaneous response, whereas approaches combining photoprotection, control of oxidative stress, modulation of inflammation, promotion of inflammatory resolution, and regulation of neuroimmune interactions may offer greater therapeutic potential. In this context, further investigation of the interactions among these systems may contribute to the identification of novel molecular targets and to the development of more effective strategies for protecting and restoring UVB-exposed skin.

## Figures and Tables

**Table 1:** Non-exhaustive summary of mechanism-based therapeutic strategies against UVB-induced skin damage.

Strategy	Representative Agents/Targets	Mechanism of Action	Key References
Antioxidant—Natural compounds	Vitamins C/E, quercetin, dieckol, ginseng phenolics, *Cordia verbenacea*	ROS scavenging; MMP inhibition; collagen preservation; NF-κB/AP-1 suppression	[73,74,75,76,77]
Antioxidant—Nrf2 activation	Sulforaphane, curcumin, flavonoids	Induction of HO-1, SOD, GST; enhancement of endogenous antioxidant capacity	[78,79]
Antioxidant—Advanced delivery	Quercetin microcapsules, NLCs, polydopamine NPs, exosome-like NPs	Improved stability, skin penetration, and controlled release of bioactives	[75,80,81,82]
Anti-inflammatory—Natural compounds	Resveratrol, naringenin, hesperidin MC, trans-chalcone.	NF-κB/COX-2 inhibition; cytokine (TNF-α, IL-1β, IL-6) suppression; oxidative stress reduction	[75,78,83,84,85]
Pro-resolving mediators (SPMs)	Resolvin D2, AT-RvD1, Lipoxin A4, Maresin 1; aspirin (via FPR2)	Active resolution of inflammation; leukocyte clearance; NF-κB downregulation; oxidative stress attenuation	[3,19,46,86,87]
Photoprotection—DNA repair	Photolyases, endonucleases (topical)	Direct reversal of CPDs and 6-4 PPs; reduction of UV-induced mutagenesis	[88]
Photoprotection—Matrix preservation	Dieckol, ginseng phenolics, quercetin	MMP-1/3 inhibition; collagen synthesis promotion; epidermal barrier restoration	[75,76,77]
Neuroimmune modulation	CGRP antagonists (microneedles), TRPV1 modulators, β-endorphin, CB1/CB2 agonists	Reduction of neuropeptide release; macrophage deactivation; NF-κB/mTOR inhibition; barrier protection	[30,89,90,91]

NLCs: nanostructured lipid carriers; NPs: nanoparticles; MC: methyl chalcone; CPDs: cyclobutane pyrimidine dimers; 6-4 PPs: 6-4 photoproducts; AT-RvD1: aspirin-triggered resolvin D1.

## Data Availability

No new data were created or analyzed in this study. Data sharing is not applicable.

## Abbreviations

The following abbreviations are used in this manuscript:

AP-1	Activating protein-1
ACE	Angiotensin-converting enzyme
AT-RvD1	Aspirin-triggered Resolvin D1
CGRP	Calcitonin gene-related peptide
Ca^2+^	Calcium
CAT	Catalase
cis-UCA	Cis-urocanic acid
cAMP	Cyclic adenosine monophosphate
CPDs	Cyclobutane pyrimidine dimers
COX-2	Cyclooxygenase-2
DAMPs	Damage-associated molecular patterns
DPP-4	Dipeptidyl peptidase 4
DRG	Dorsal root ganglia
ET-1	Endothelin-1
ERK	Extracellular Signal-Regulated Kinase
GSTs	Glutathione S-transferases
G-CSF	Granulocyte colony-stimulating factor
HO-1	Heme oxygenase-1
FcεRI	High-affinity receptor for immunoglobulin E
H_2_O_2_	Hydrogen peroxide
IL-1β	Interleukin-1 beta
IL-1	Interleukin 1
IL-2	Interleukin 2
IL-4	Interleukin 4
IL-6	Interleukin 6
IL-8	Interleukin 8
IL-10	Interleukin-10
IL-17	Interleukin-17
JAKs	Janus tyrosine kinases
Keap1	Kelch-like ECH-associated protein 1
LPO	Lipid peroxidation
MMP-1	Matrix metalloproteinase-1
MMP-9	Matrix metalloproteinase-9
MITF	Microphthalmia-associated transcription factor
MAPK	Mitogen-activated protein kinase
MyD88	Myeloid differentiation primary response 88
NOX	NADPH oxidases
JNK	N-terminal kinase c-Jun
NGF	Nerve growth factor
NEP	Neutral endopeptidase
Nrf2	Nuclear factor erythroid 2-related factor 2
NF-κB	Nuclear factor kappa B
ROS	Reactive oxygen species
PI3K	Phosphoinositide 3-kinase
PGE2	Prostaglandin 2
NLRP3	Pyrin domain containing protein 3
GSH	Reduced glutathione
5-HT	Serotonin
STATs	Signal transducer and transcription-activating proteins
SALT	Skin-associated lymphoid tissue
SP	Substance P
O_2_•^−^	Superoxide anion
SOD	Superoxide dismutase
TLR4	Toll-like receptor 4
TLRs	Toll-like receptors
trans-UCA	Trans-urocanic acid
TEWL	Transepidermal water loss
TRPA1	TRP ankyrin 1
TRPM8	TRP melastatin 8
TRPV1	TRP vanilloid 1
TRPV4	TRP vanilloid 4
TNF-α	Tumor necrosis factor-alpha
TRAF6	Tumor necrosis factor receptor-associated factor 6
TRP2	Tyrosinase-related protein 2
UVR	Ultraviolet radiation
XPA	Xeroderma Pigmentosum group A
α-MSH	Alpha-melanocyte-stimulating hormone
L-DOPA	3,4-Dihydroxy-L-phenylalanine
I-RTX	5′-Iodoresiniferatoxin
